# Diversity and composition of the *Panax ginseng* rhizosphere microbiome in various cultivation modesand ages

**DOI:** 10.1186/s12866-020-02081-2

**Published:** 2021-01-08

**Authors:** Ai-Zi Tong, Wei Liu, Qiang Liu, Guang-Qing Xia, Jun-Yi Zhu

**Affiliations:** 1 School of Life Science, Tonghua Normal University, No.950 Yu Cai Road, Dongchang District, Tonghua, 134002 China; 2 Chinese Institute of Jilin Ginseng, Tonghua, 134002 China

**Keywords:** Cultivation ages, Cultivation modes, *Panax ginseng*, Rhizosphere microbiome

## Abstract

**Background:**

Continuous cropping of ginseng (*Panax ginseng* Meyer) cultivated in farmland for an extended period gives rise to soil-borne disease. The change in soil microbial composition is a major cause of soil-borne diseases and an obstacle to continuous cropping. The impact of cultivation modes and ages on the diversity and composition of the *P. ginseng* rhizosphere microbial community and technology suitable for cropping *P. ginseng* in farmland are still being explored.

**Methods:**

Amplicon sequencing of bacterial 16S rRNA genes and fungal ITS regions were analyzed for microbial community composition and diversity.

**Results:**

The obtained sequencing data were reasonable for estimating soil microbial diversity. We observed significant variations in richness, diversity, and relative abundances of microbial taxa between farmland, deforestation field, and different cultivation years. The bacterial communities of LCK (forest soil where *P. ginseng* was not grown) had a much higher richness and diversity than those in NCK (farmland soil where *P. ginseng* was not grown). The increase in cultivation years of *P. ginseng* in farmland and deforestation field significantly changed the diversity of soil microbial communities. In addition, the accumulation of *P. ginseng* soil-borne pathogens (*Monographella cucumerina*, *Ilyonectria mors*-*panacis*, *I. robusta*, *Fusarium solani*, and *Nectria ramulariae*) varied with the cropping age of *P. ginseng*.

**Conclusion:**

Soil microbial diversity and function were significantly poorer in farmland than in the deforestation field and were affected by *P. ginseng* planting years. The abundance of common soil-borne pathogens of *P. ginseng* increased with the cultivation age and led to an imbalance in the microbial community.

**Supplementary Information:**

The online version contains supplementary material available at 10.1186/s12866-020-02081-2.

## Introduction

As a traditional Chinese herbal medicine, the perennial plant ginseng (*Panax ginseng* Meyer) is famous for its benefits to the immune system [1, 2], nervous system [3], cardiovascular system [4–6], and has an anti-cancer effect [7, 8]. *P. ginseng* is mainly grown in Asia, particularly in China, Korea, and Russia [9]. China has a long history of *P. ginseng* cultivation. In particular, *P. ginseng* produced from the Changbai Mountain area in eastern Jilin Province in China is well-known for its high yield and top quality [10]. For the past 400 years, *P. ginseng* has been mainly cultivated in the deforestation field. However, this cultivation method was banned by the Chinese government because of its negative impact on forest resources and the ecological environment. Currently, the cultivation is mainly conducted in the farmland, which has led to serious soil-borne diseases of *P. ginseng*. Consequently, the yield and quality of *P. ginseng* cannot be guaranteed, causing huge economic losses and impeding the healthy and sustainable development of the national ginseng industry.

Soil microorganisms are responsible for the decomposition and cycling of organic compounds [11]. They also influence above-ground ecosystems by contributing to plant nutrition and health [12], and soil structure and fertility [13]. Rhizospheric microorganisms buffer the effects of toxic compounds in the soil and soil-borne pathogens [14]. Therefore, the soil microbial community is an important biological indicator of soil functions [15, 16]. The structure and relative activity of microbial communities are often different across plant species, cultivation years, and plant developmental stages [17]. A few recent studies on the association between continuous cropping and microbial community revealed that continuous farming led to alterations in microbial community diversity of rhizospheric soils [18–20]. For instance, compared with traditional crops, the diversity and composition of soil microbiome changed in soil with continuously planted American ginseng [20]. A significant change was also observed in the microbial communities of the arable soil with continuously planted *P. ginseng* [21]. In addition, different planting ages of *P. ginseng* could also alter soil microbial community [22].

*P. ginseng* grows continuously in fixed plots for 4 to 5 years. The soil-borne diseases of *P. ginseng* become more serious with the plant ages, causing root rot and rusty root, and reducing the survival rate of *P. ginseng*. Soil-borne diseases may be influenced by the changes in soil microbial communities [23]. Accordingly, a direct association was observed between the prevalence of *P. ginseng* soil-borne diseases in farmlands and the pathogen accumulation [16]. Understanding the diversity and composition of microbial communities in different types of soil, and the relationship between microorganisms and cultivation ages of their host plants, such as *P. ginseng*, could be helpful in the prevention and control of soil-borne diseases and increase productivity. While polymerase chain reaction denaturing gradient gel electrophoresis and random amplification of polymorphic DNA methods are used to analyze the microbial diversity of *P. ginseng* rhizosphere soil at different ages and cultivation modes [22, 24], most microbes are still difficult to be analyzed using these traditional methods. Compared with traditional methods of analysis, high-throughput sequencing technology is one of the most widely used tools for evaluating *P. ginseng* rhizospheric bacterial and fungal community structure. In this study, we carried out amplicon sequencing to analyze the diversity and structure of *P. ginseng* rhizospheric microbial communities from farmland and deforestation field soil during zero to 5 y of *P. ginseng* cultivation. This study allowed us to evaluate the effects of the two land types and cultivation years on the microbial communities.

## Results

### Amplicon sequencing and rarefaction curves

To characterize the microbiome in *P. ginseng* rhizospheric soil at different cultivation ages under two land types, 36 samples were sequenced by Illumina MiSeq. The amplicon sequencing resulted in a total of 1,362,294 reads of bacterial 16S rRNA genes, and 1,355,565 reads of fungal ITS regions after quality trimming and assigning. The average lengths of the obtained bacterial and fungal sequence were 436 bp and 263 bp, respectively. High-quality sequences with 97% sequence similarity were gathered into 4061 bacterial OTUs and 3102 fungal OTUs.

**Fig. 1:**
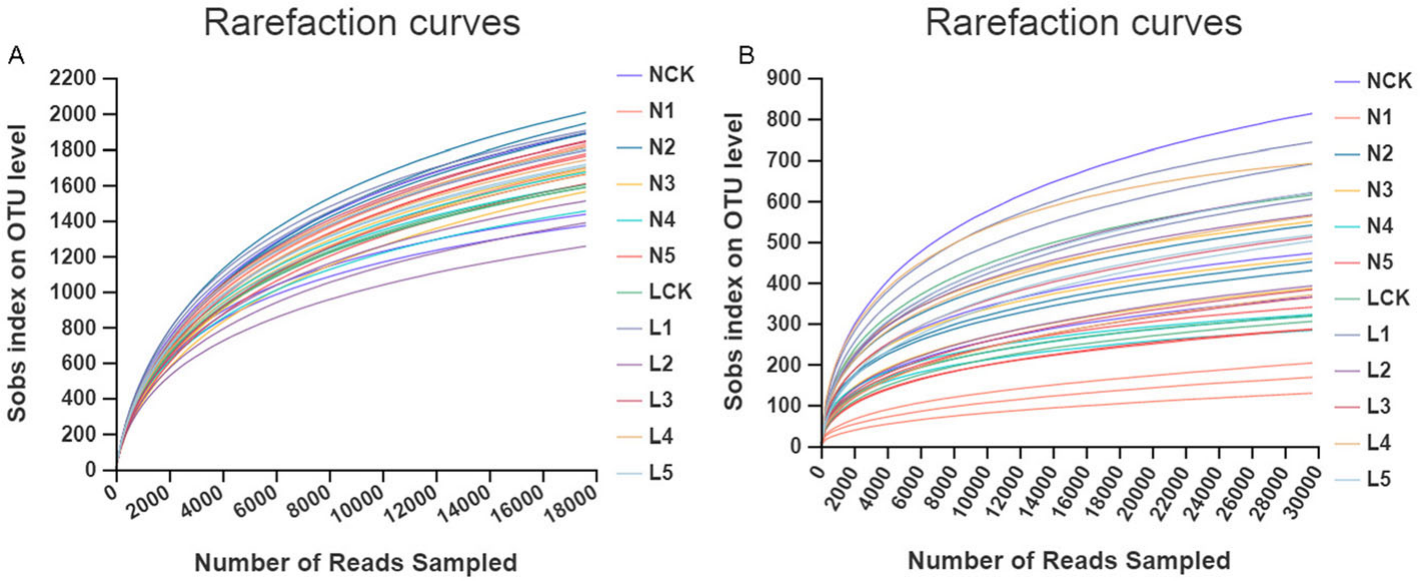
Rarefaction curves of individual soil samples (**a**. bacteria, **b**. fungi).The rarefaction curve was assembled using the Sobs index at the OTU level. Relative to the total number of sequences, sequence similarity was defined at 97% cut-off by Mothur

To construct rarefaction curves, the dataset was flattened according to the minimum number of sequence of the samples. The rarefaction curves were constructed based on the number of OTUs observed in the individual sample (Fig. 1). The rarefaction curves for evaluating the richness of each sample usually approached saturation, indicating that the sequencing data were reasonable for evaluating the microbial diversity of *P. ginseng* rhizosphere. The fungal rarefaction curves exhibited a higher degree of variation in shape compared to that of bacterial OTUs.

### Alpha diversity of bacterial and fungal communities

**Fig. 2:**
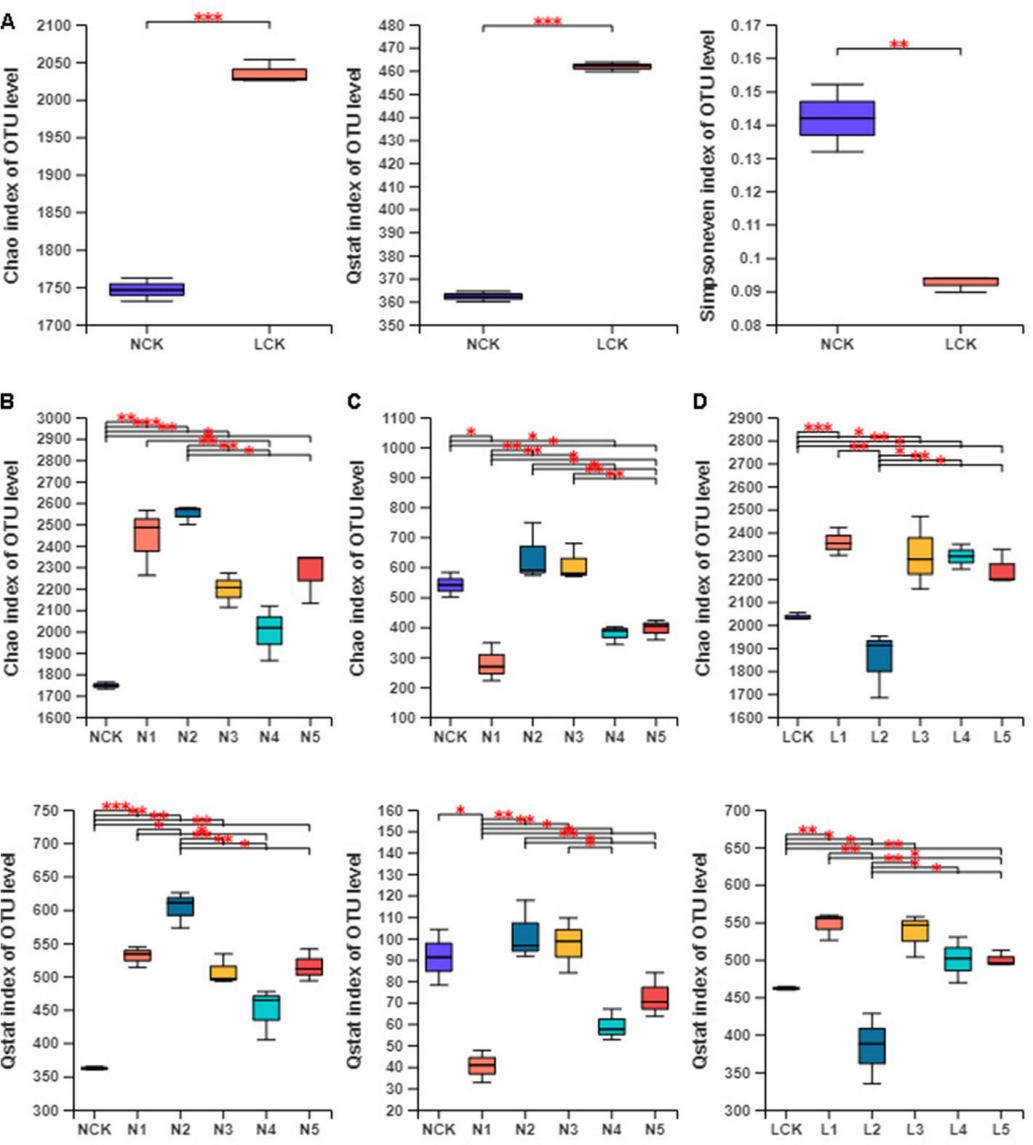
Estimate the microbial community by alpha diversity. **a**, Alpha diversity of bacterial communities between NCK and LCK. **b**, Alpha diversity of bacterial communities among NCK, N1, N2, N3, N4, and N5. **c**, Alpha diversity of fungal communities among NCK, N1, N2, N3, N4, and N5. **d**, Alpha diversity of bacterial communities among LCK, L1, L2, L3, L4, and L5. Estimation of Alpha diversity representing two biological replicates for the NCK samples, and three biological replicates for the other rhizospheric soil samples (*p < 0.05, **p < 0.01, ***p < 0.001)

To estimate differences in the alpha diversity, we trimmed-off the minimum number sequence from samples of the dataset. Pairwise hierarchical clustering using Bray-Curtis dissimilarity revealed that the sample NCK3 was distinctly separated from NCK1 and NCK2. Therefore, we removed the NCK3 data from analyzing the alpha diversity. The OTU Chao richness index, the Simpsoneven evenness index, and the Q statistics of species diversity (Qstat) were used to reflect the alpha diversity of microbial communities in *P. ginseng* rhizosphere (Fig. 2). First, the alpha diversity of the microbial community was evaluated in the farmland and deforestation field with no *P. ginseng* planting. As expected, the bacterial communities of the deforestation field (LCK, forest soil where *P. ginseng* was not grown) had a much higher richness and diversity than those of the farmland (NCK, farmland soil where *P. ginseng* was not grown) (Fig. 2a). OTU richness value of LCK (2035.1 ± 15.8) was higher than that of NCK (1746.3 ± 21.6) and separated from NCK (*P* < 0.001). On the contrary, the LCK had a lower OTU evenness than that of NCK (*P* < 0.01). However, NCK and LCK did not exhibit variations in the richness, evenness, and diversity of the fungal communities (Fig. S1A).

*P. ginseng* is a perennial herb and that needs to grow continuously in the same place for many years. We observed that the alpha diversity of the soil microbial community changed significantly with the cultivation years of *P. ginseng* in farmland and deforestation field. The farmland and the deforestation field exhibited similar patterns of soil bacterial community richness and diversity in terms of the cultivation years (Fig. 2b, d). The richness and diversity of farmland soil bacteria showed an increasing trend in the first 2 y of *P. ginseng* planting and significantly decreased in the third year. However, in the first 3 y of planting *P. ginseng* in the forest field, the bacterial richness and diversity first increased, then decreased, and increased again. In the fourth and fifth years of *P. ginseng* cultivation in both farm land and deforestation field, the bacterial richness and diversity were stable relative to those in the third year. To sum up, after growing *P. ginseng* continuously for five years (N5/L5), the richness and diversity of soil bacteria increased compare to those in NCK or LCK. After *P. ginseng* cultivation in the farmland, fungal richness and diversity decreased in the first year, increased in the second and third years, and decreased again in the fourth and fifth years. The richness of the fungal community decreased in farmland soil after growing *P. ginseng* for five years (N5) compared to that in NCK, although the diversity did not change significantly (Fig. 2c). The fungal community richness and diversity in forest soil were less affected by the number of cultivation years and showed no marked variation in the richness and diversity after growing *P. ginseng* for five years (L5), compared with those in LCK (Fig. S1B).

### Beta diversity of bacterial and fungal communities

**Fig. 3:**
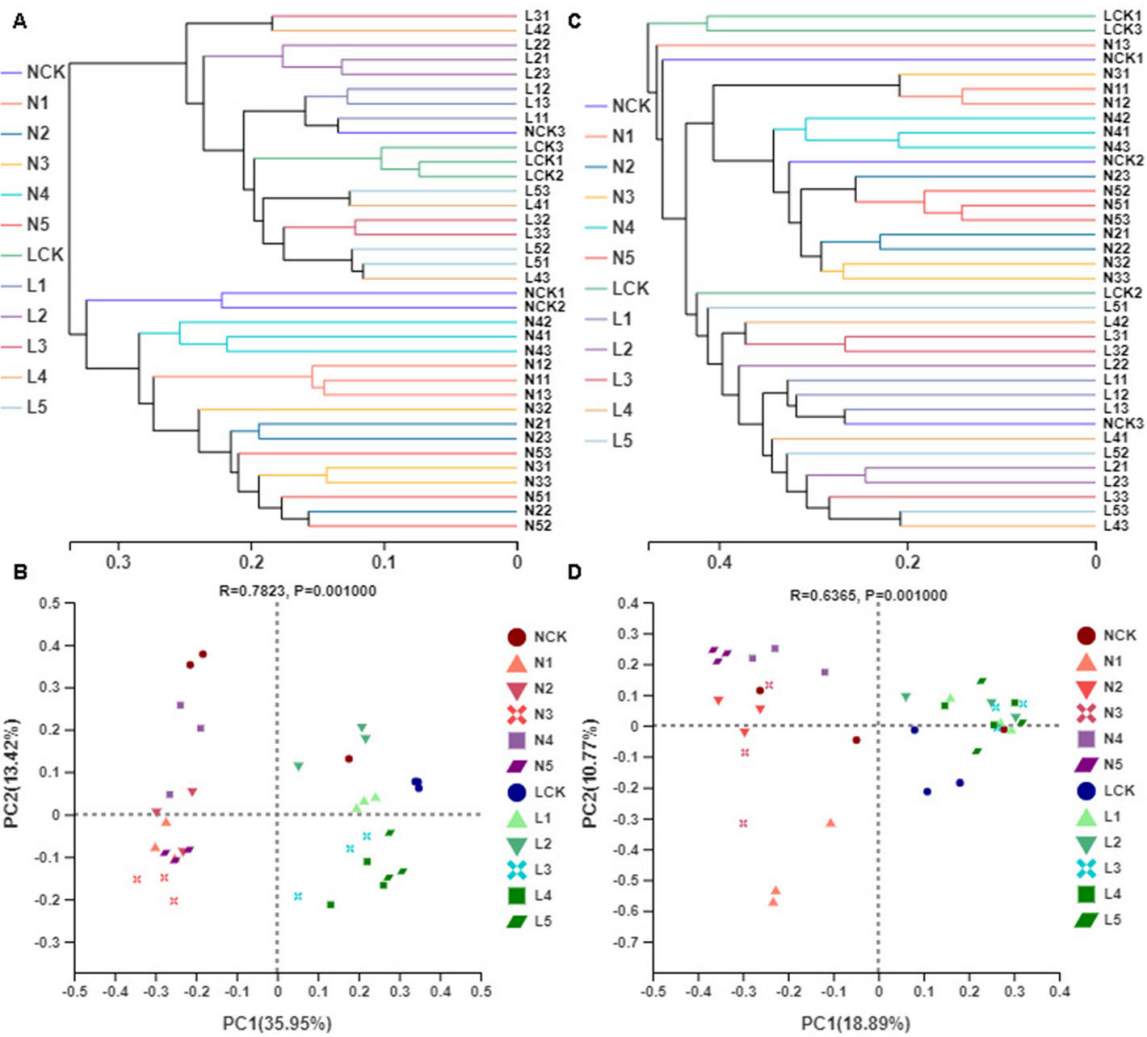
Estimation of similarity and distance based on the hierarchical clustering tree of the samples (**a**, bacteria; **c**, fungi) and principal coordinate analysis (PCoA) (**b**, bacteria; **d** fungus) of the OTUs. The length of the branches in the hierarchical tree represents the distance between samples. The closer the two samples are on the PCoA plot, the more similar the species composition of the two samples

We estimated beta diversity based on phylogenetic levels of OTUs of bacterial and fungal communities. The community composition in different samples was compared using the Bray-Curtis distance matrix and the primary factors driving the community composition were determined by calculating the abundance of reads after normalization and square root conversion. Hierarchical clustering was constructed based on Bray-Curtis dissimilarities. Principal coordinate analysis (PCoA) was performed to determine the overall similarity of microbial community structure among samples; when combined with the PERMANOVA analysis, it showed a significantly different community composition of bacteria (R^2^ = 0.711, *p* = 0.001) and fungi (R^2^ = 0.562, p = 0.001) in different sample groups. The farmland soil samples, except for NCK3, were clearly distinct from those of deforestation field, farmland, and forest soil samples (Fig. 3).

The hierarchical clustering of pairwise Bray–Curtis dissimilarities of bacterial OTUs revealed complete clustering according to the rhizosphere soil indifferent fields but did not cluster completely according to their cultivation years (Fig. 3a). PCoA analysis revealed that the horizontal coordinate was the primary coordinate component that caused differences in the composition of microbial community indifferent soil samples. In terms of the OTUs, the variance contribution rates of the PC1 to the difference in bacterial and fungal community composition of samples were 35.95 and 18.89%, respectively (Fig. 3b, d).

### The composition and structure of the bacterial community

**Fig. 4:**
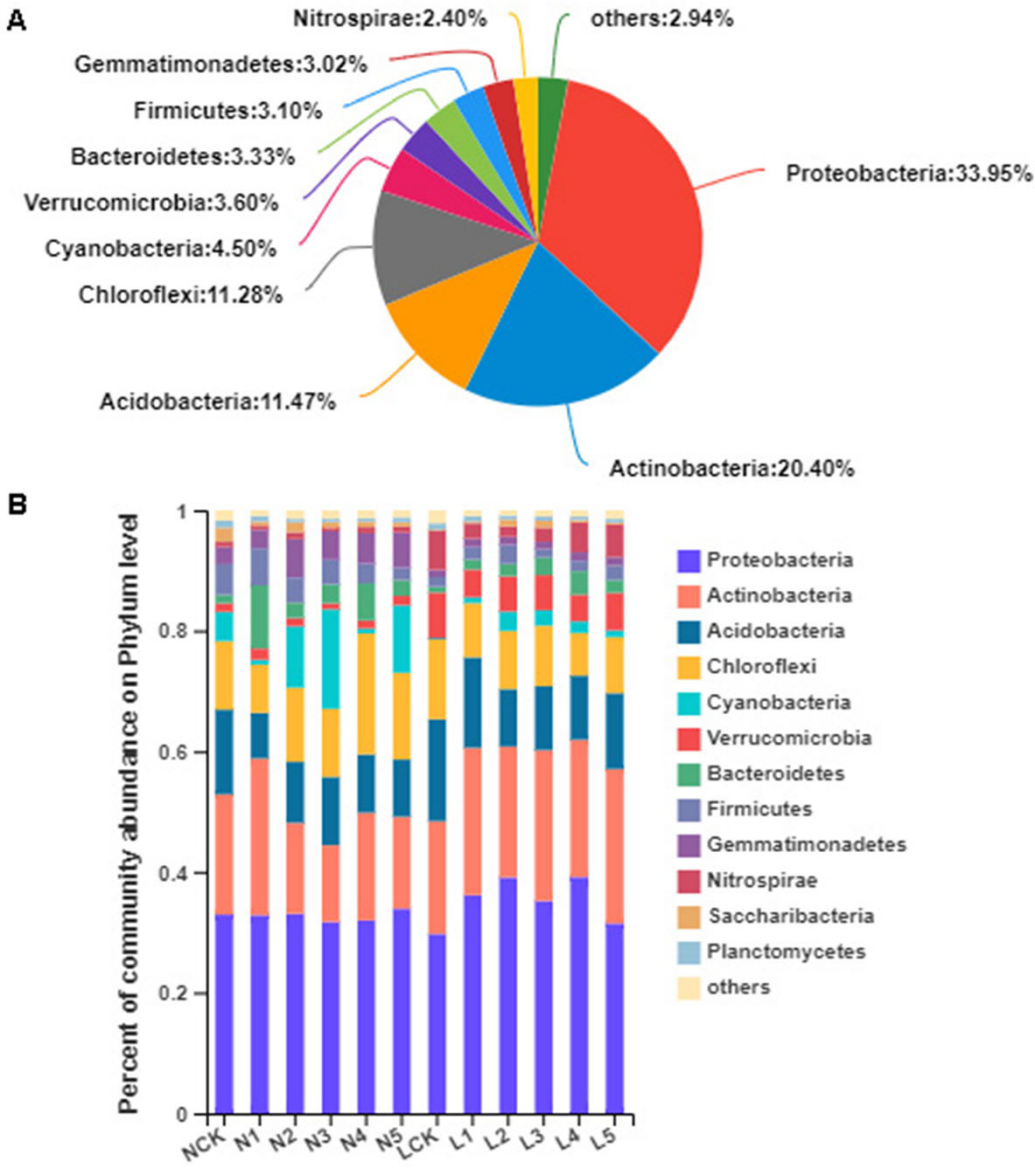
The composition and structure of the bacterial community. A, Pieplot for bacterial community analysis at the phylum level. B, Relative abundances of the bacterial phyla in different samples

From bacterial 16S rRNA gene sequences, 36 prokaryoticphyla, 89 classes, 180 orders, 346 families, 656 genera, 1439 species, and 4056OTUs were identified based on 97% species similarity. The bacterial phyla Proteobacteria, Actinobacteria, Acidobacteria, and Chloroflexi were the richest in both the farmland or deforestation field under *P. ginseng* cultivation (Fig. 4a). We analyzed the dominant bacterial phyla and observed distinct bacterial communities in the two types of lands. Continuous cultivation years of *P. ginseng* in the same field also drastically affected the soil microbial community structure. The first 15 phyla were assessed using ANOVA to test the impact of different lands (NCK vs LCK, N5 vs L5) and cultivation years (NCK vs N5, LCK vs L5) on their relative abundance (%) (Fig. 4 B). With the exception of Proteobacteria (*P* = 0.274), Cyanobacteria (*P* = 0.068), and Planctomycetes (*P* = 0.400), the top 15 relatively abundant bacterial phyla were significantly different among different samples (Fig. S2). In the LCK soil samples, we found significant enrichment (*P* < 0.05) of Verrucomicrobia, Nitrospirae, Latescibacteria, and Tectomicrobia (relative abundance = 7.64,6.48, 0.75, and 0.42%), compared to those in the NCK. The significant difference between the forest field (the L group) and the farmland (the N group) were further assessed by Student’s t-test. The relative abundances of Actinobacteria (23.03%), Verrucomicrobia (5.77%), Nitrospirae (3.92%), and Latescibacteria (0.35%) were remarkably increased in the forest field as compared to those in the farmland, while Chloroflexi (9.70%), Cyanobacteria (1.64%), Bacteroidetes (2.27%), Firmicutes (2.07%), Gemmatimonadetes (1.35%), Saccharibacteria (0.57%), and Parcubacteria (0.09%) were decreased in relative abundances. During the first year of *P. ginseng* cultivation in the farmland, the relative abundance of Acidobacteria (7.56%) decreased compared to that in NCK (14.07%). The relative abundance of Actinobacteria decreased in N2 (15.03%) compare to N1 (25.98%), while that of Parcubacteria increased in bothN1 and N2 (0.04 and 0.28%, respectively). The relative abundance of Bacteroidetes in N1 (10.49%) increased compared to that in NCK (1.41%) and N2 (2.55%). The relative abundance of Cyanobacteria decreased in N4 (0.81%) compared to that in N3 (16.46%), while that of Nitrospirae increased in both N3 and N4 (0.56 and 0.99%, respectively). After five years of *P. ginseng* cultivation in farmland, the relative abundance of Acidobacteria (9.52%) decreased significantly in N5 as compared to that in NCK, while the relative abundance of Bacteroidetes (2.62%) increased (Fig. S3A).

In the first year of *P. ginseng* cultivation in the deforestation field, the relative abundance of Proteobacteria (LCK 29.75%, L1 36.19%), Actinobacteria (LCK 18.64%, L1 24.41%), and Saccharibacteria (LCK 0.16%, L1 0.29%) increased, while that of Chloroflexi (LCK 13.27%, L1 9.02%), Latescibacteria (LCK 0.75%, L1 0.19%), and Tectomicrobia (LCK 0.42%, L1 0.10%) decreased. The relative abundance of Acidobacteria in L2 was less than that in L1 (9.54, and 14.95%, respectively), while that of Saccharibacteria increased in both L1 and L2 (0.29, and 1.16%, respectively). The relative abundance of Firmicutes in L3 was less than that in L2 (1.41 and 3.18%, respectively). The relative abundance of Tectomicrobia was higher in L4 than that inL3 (0.29 and 0.14%, respectively). After five years of continuous cultivation of *P. ginseng* in the forest field, there was a remarkable increase in the relative abundances of Actinobacteria (25.58%), Bacteroidetes (2.14%), and Cyanobacteria (1.11%) in the L5 as compared with those in the LCK. Finally, the relative abundances of Actinobacteria (25.58%), Nitrospirae (5.48%), and Tectomicrobia (0.38%) increased noticeably in L5 than inN5, while that of Gemmatimonadetes (1.41%) decreased (Fig. S3B).

### The composition and structure of the fungal community

**Fig. 5:**
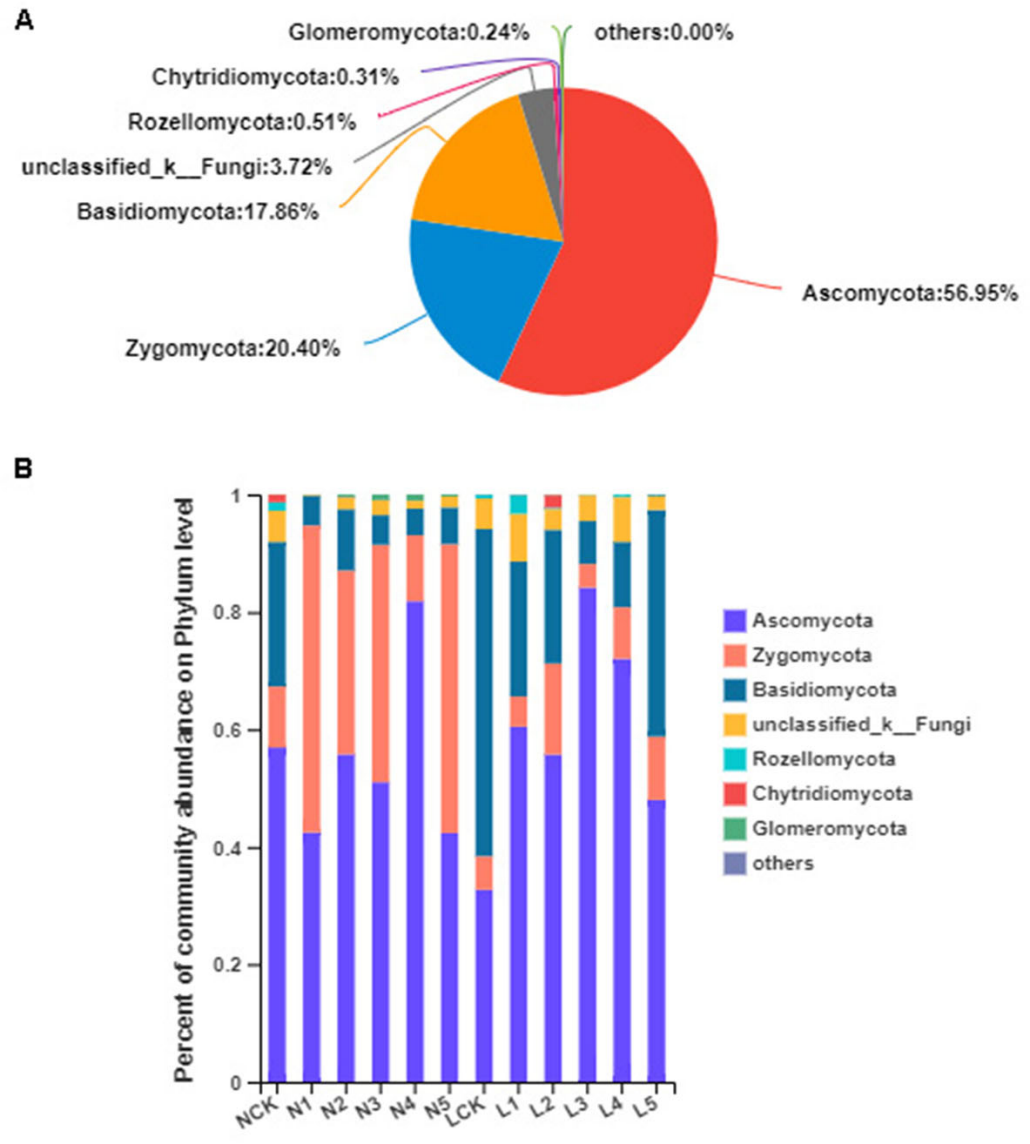
The composition and structure of the fungal community. A, Pieplot for fungal community analysis at the phylum level. B, Relative abundances of the fungal phylain different samples

From fungal ITS sequences,eighteukaryoticphyla,31 classes, 104 orders,231 families,517 genera, 894species, and 3088 OTUs were identified. Fungal OTUs primarily consisted of phyla Ascomycota, Zygomycota, Basidiomycota, Rozellomycota, Chytridiomycota, and Glomeromycota. Ascomycota was the most abundant in the farmland as well as in the deforestation field during the cultivation of *P. ginseng* (Fig. 5a). We assessed the dominant fungal phyla using ANOVA, which distinguished the fungal communities in two different soil types and cultivation years based on their relative abundance (%) (Fig. 5b).The relative abundance of Ascomycota and Zygomycota varied significantly (*P* < 0.05) on different soils and cultivation years among nearly all identified fungal phyla (Fig. S4). Compared to the NCK, no significant divergence of eukaryoticphyla was observed in the LCK samples. The student’s t-test was performed to analyze any significant difference between the fungal communities in the forest field (group L) and the farmland (group N).The relative abundances of Zygomycota and Glomeromycota increased significantly in the farmland (32.46and 0.46%, respectively) as compared to those in the forest field (8.33and 0.03%, respectively), while that of Basidiomycota (9.34and26.39%, respectively) decreased (Fig. S5). In the second year of *P. ginseng* cultivation, there was an increase in the relative abundances of Glomeromycota and Rozellomycota in N2 (0.38 and 0.02%, respectively) compared to those in the N1 soil sample, which was 0% for both phyla. The relative abundance of Rozellomycota in N3 (0%) decreased compared to that in N2 (0.02%), and was nearly similarbetweenN3 and N4, and between N4 and N5. After growing *P. ginseng* for five years in the farmland, the relative abundance of Zygomycota (49.16%) increased remarkably in N5 compared to that in NCK (Fig. S5). In different years of continuous *P. ginseng* plantation in the forest field, the variations in the relative abundances of eukaryoticphyla were relatively small. The relative abundance of Ascomycotain L1 increased remarkably (60.46%) compared to that in LCK (32.71%). The relative abundance of Zygomycota in L2 increased (15.53%) compared to that in L1 (5.15%) and L3 (3.96%) and was nearly similar between L3 and L4, and between L4 and L5. After planting *P. ginseng* for five years in the forest field, the relative abundances of eukaryotic phyla did not vary significantly in L5 as compared to that in LCK. Finally, the relative abundance of Zygomycota was remarkably higher in N5 (49.16%) than that in L5 (10.83%) (Fig. S5).

### Pathogenic fungal enrichment varies with *P. ginseng* cropping age

**Fig. 6:**
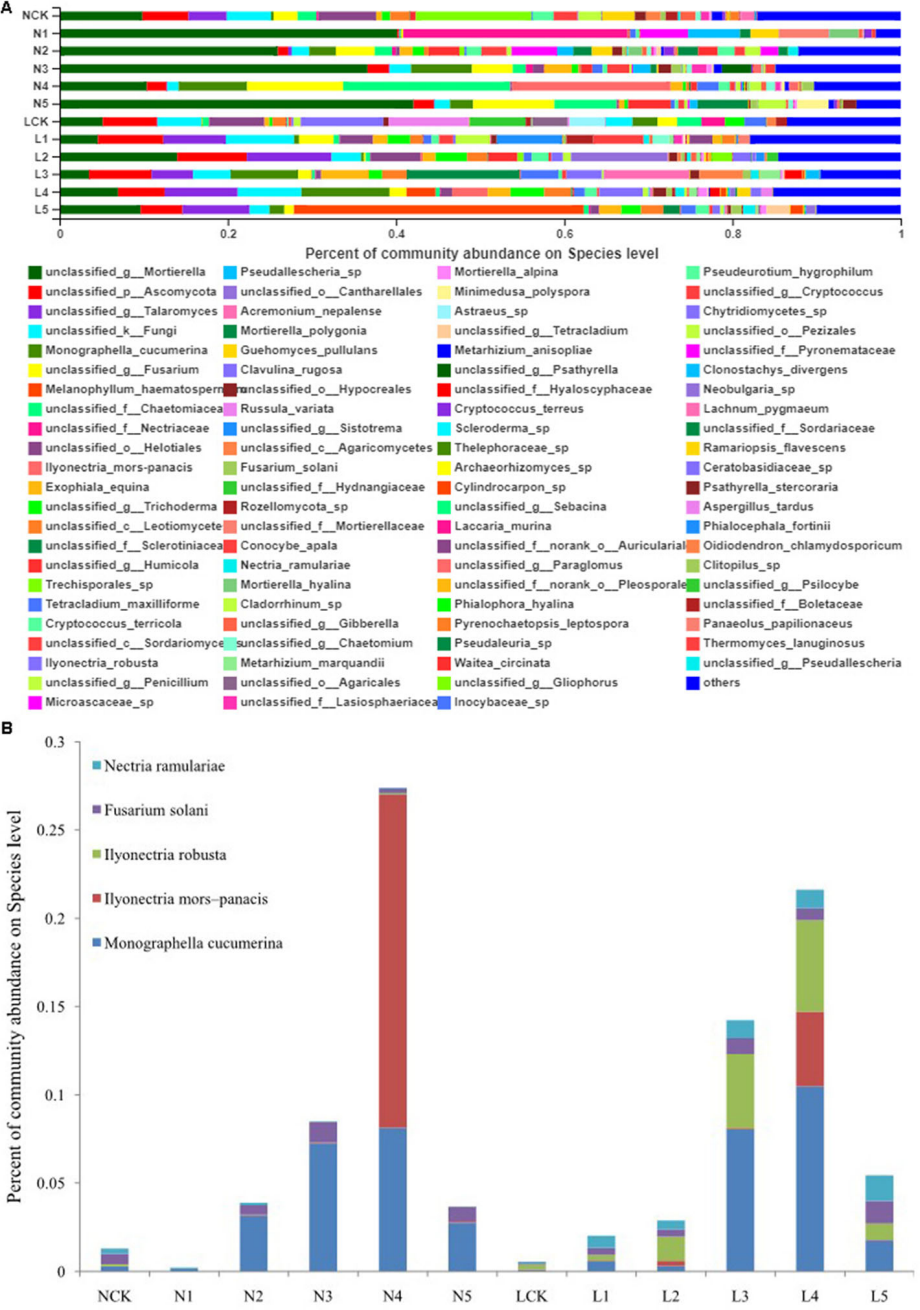
The relative abundance of pathogenic fungal in soil samples. Three biological replicates of each sample. A, Relative abundances of the different fungal in different samples at genus level. B, Relative abundance of pathogenic fungal species in soil samples

With the increase in the number of cultivation years of *P. ginseng*, its soil-borne diseases become progressively serious. Therefore, it was speculated that soil-borne pathogens accumulate with the increase of *P. ginseng* cropping years. *Mortierella* was the most abundant genus in the tested soil samples (Fig. 6a). *Fusarium* is a common pathogenic microorganism that causes soil-borne diseases in plants. In our study, *Fusarium* was estimated to be the third most abundant genus, which was much higher in farmland soil than in the forest soil. *Gibberella*, the perfect stage of Fusarium, was also detected in the samples [25]. In addition to ginseng root rot, another major *P. ginseng* soil-borne disease is ginseng rust rot, which is caused by *Cylindrocarpon*. We observed similar distribution trends of *Cylindrocarpon* and *Nectria*. *Nectria* belongs to Nectriaceae and is the perfect stage of *Cylindrocarpon*. With the cropping of *P. ginseng*, the abundances of *Cylindrocarpon* and *Nectria* increased and were the highest in L5 soil samples.

Accordingly, we further analyzed the changes in the abundances of pathogenic species. We found that five pathogenic species (*Monographella cucumerina*, *Ilyonectria mors*-*panacis*, *I. robusta*, *Fusarium solani*, and *Nectria ramulariae*) accumulated differently in *P. ginseng* rhizosphere soil samples of different plantation years and patterns (Fig. 6b). *M. cucumerina* significantly accumulated in the second year of *P. ginseng* planting and increased annually, reaching its maximum value in the fourth year in the farmland (8.14%).The accumulation of *M. cucumerina* increased significantly in the third and fourth years of planting *P. ginseng* in the forest field (8.05 and 10.48%,respectively).*I. mors*-*panacis* accumulated significantly in the fourth year of planting *P. ginseng*, in the farmland as well as in the forest field (18.84 and 4.23%,respectively). *I. robusta* accumulated more in forest land than in farmland, especially from the second year of planting *P. ginseng*, and its accumulation increased annually, reaching the maximum in the fourth year (5.18%). *F. solani* accumulated more in NCK (0.59%) than in LCK (0.09%), with the maximum found in the forest land with *P. ginseng* cultivation. *N. ramulariae* accumulated more in NCK (0.29%) than in LCK (0.08%), and its abundance decreased in the farmland with *P. ginseng* planting, but increased in the forest land, reaching the highest in the fifth year (1.45%). In short, the accumulation of *P. ginseng* soil-borne pathogens varied with the cropping ages of *P. ginseng* as observed in our study.

## Discussion

Soil-borne diseases are common and serious problems faced in the *P. ginseng* cultivation, especially in the farmlands. The soil fertility in farmlands with continuous planting of crops is maintained by increasing the use of chemical fertilizers, which reduces beneficial microorganisms and increases the content of soil-borne pathogens each year. The relationship between soil-borne diseases and soil microbiome under the continuous *P. ginseng* cultivation in farmlands seems to be important for epidemiological studies. In this study, we found that the *P. ginseng* cultivation, especially on different lands and for different durations, affects the diversity and structure of the microbial community in the *P. ginseng* rhizosphere, in accordance with findings from previous studies [24, 26]. Imbalances in rhizospheric microbial communities disrupt *P. ginseng* cultivation [24].

Soil microorganisms play a vital role in maintaining the stability and health of the soil ecological systems. Variations in the microbial community may adversely affect soil quality and plant health. Hence, it is important to study the variation in the soil microbial community during *P. ginseng* cultivation. The microbial community can be used as a sensitive biological indicator of soil health and function [15] and associates well with root disease suppression [27]. In this study, the richness and diversity of bacterial communities in forest soils without *P. ginseng* plantation (LCK) were significantly higher than in farmland soils (NCK) (Fig. 2a), suggesting a better soil function in the deforestation field.

The developmental stage of a plant is an important driving force for microbial community structure [17]. The richness and diversity of microbial communities (including bacteria and fungi) in farmland were found to change significantly with the cropping years of *P. ginseng* (Fig. 2b). The diversity of bacteria in the farmland increased after *P. ginseng* planting, while the fungal diversity decreased significantly in the first year of *P. ginseng* planting, compared to that in NCK. However, the variation trend of bacterial richness and diversity in the forest soil after planting *P. ginseng* was similar to that of the farmland. It changed significantly in the first three years of planting *P. ginseng*, and gradually stabilized in the fourth and fifth years. The richness and diversity of fungi were less affected by the number of years of *P. ginseng* planting (Fig. S1B). During the first to third years, *P. ginseng* is in the seedling stage; at this stage, the soil microbial community changed significantly and showed an undulating trend. In the maturity stage (the fourth to sixth years), when *P. ginseng* is ready to be harvested, the soil microbial community tended to stabilize.

The soil samples of different ages of *P. ginseng* planting were obtained from different locations, but the pattern of microbial community composition was similar in the locations with the same planting pattern. However, the microbial community composition of soil samples from the two different planting modes was significantly different. The bacterial phyla Proteobacteria, Actinobacteria, Acidobacteria, and Chloroflexi, and the fungal phylum Ascomycota were the most abundant during *P. ginseng* cultivation, indicating their widespread presence in the soil ecological system of *P. ginseng* monoculture, which is consistent with previous reports on cotton [18], soybean [28], and peanut [29] under continuous planting regimes. In the bacterial communities, the ratio of Proteobacteria to Acidobacteria is an index of soil nutrients, in which Proteobacteria is associated with eutrophic soil, and Acidobacteria is associated with infertile soil [30, 31]. Proteobacteria plays a crucial role in the global carbon, nitrogen, sulfur, and iron cycles [32, 33], and was the most abundant (Fig. 5a), which is consistent with findings of previous studies [34–36]. Actinobacteria was the second most abundant; it participates in the global carbon cycling [37] and the decomposition of soil organic material [38]. In an earlier study, Actinobacteria was used as a bio-control agent to control plant diseases transmitted by soil and seeds [39]. Similar to our findings, the primary bacteria in *P. ginseng* soil were Proteobacteria and Actinobacteria [26]. Interestingly, the abundance of Actinomyces varied with the *P. ginseng* cultivation, indicating that the *P. ginseng* cropping remarkably influenced the community of Actinobacteria. Therefore, the members of both Proteobacteria and Actinobacteria have a potential role in the microbial homeostasis in the continuous cropping soil.

Soil traits and crop varieties can have impact on the rhizosphere microbial community [40, 41], and the cropping year is considered to be a key factor driving this [34].The study showed that the microbiological compositions of the *P. ginseng* rhizospheric soil varied markedly with cultivation years. This phenomenon may be caused by the root exudates during the different growth stages [42–44], which can alter rhizospheric microbial community, during plant growth [40, 45]. *P. ginseng* also contains allelochemicals that can disturb the balance of microbial community [46], and reduce beneficial fungi while increase pathogenic fungi in *P. ginseng* rhizospheric soil [47].

*Mortierella* was the most abundant genus in the *P. ginseng* rhizospheric soil samples. It is widespread in the temperate zone and has not been previously reported as a *P. ginseng* pathogen. *Fusarium*, the third most-enriched genus, is a pathogen of many plants. *F. solani* was found to be the primary pathogenic species causing *P. ginseng* root rot. *F.solani* accumulated with the growth of *P. ginseng*, especially in the forest field. Furthermore, *Gibberella,* a perfect stage for *Fusarium* [25], is also an important soil-borne pathogen. Other major *P. ginseng* soil-borne pathogens, *Cylindrocarpon* and *Ilyonectria* that cause ginseng rust rot disease, also varied in abundances with the cultivation of *P. ginseng*. Currently, the isolates of *Ilyonectria* species that infect *P. ginseng* are categorized into four species: *I. robusta*, *I. mors-panacis*, *I. panacis*, and *I. crassa* [48, 49]. In this study, *I. mors-panacis* and *I. robusta* were noticeably enriched in the soil with *P. ginseng* cultivation, and the extent of accumulation varied with different cultivation years and soil types. *N. ramulariae* (anamorph: *C. obtusiusculum*) is a well-known soil-born fungus across the world [50]. Root rot of ginseng induced by *Fusarium*, *Cylindrocarpon*, and *Ilyonectria* is the main reason for the declined survival rate of *P. ginseng*. This study revealed that the total concentration of pathogenic species of *P. ginseng* increased annually during the first to fourth years of *P. ginseng* planting, reached the maximum in the fourth year, and decreased significantly in the fifth year, in farmland as well as in the forest field (Fig. 6b). *I. mors-panacis* was specifically enriched in the fourth year of *P. ginseng* growth in the farmland and the forest field, while *I. robusta* and *N. ramulariae* were specifically enriched in the forest field. *M. cucumerina*isa fungal plant pathogen, and was found to accumulate the most among the aforementioned five pathogenic species. *Monographella* increased in abundance in the rhizosphere soil of *P. quinquefolius* when root rot occurred [51, 52].

Overall, it is likely that the increase in the abundances of soil-borne pathogenic fungi and the decrease in the abundances of other non-pathogenic fungi occurred under continuous single-cropping of *P. ginseng*. When the soil microbial community is disordered and rich in pathogenic microorganisms, it becomes difficult to control root colonization and insurgence of fatal disease seven under appropriate conditions. This study offers new insights into the influences of continuous planting *P. ginseng* on rhizosphere soil microbial communities.

## Conclusions

The bacterial and fungal taxa revealed a remarkable difference in composition among the two types of fields with continuous cropping of *P. ginseng*. These effects jointly lead to disturbed microbial communities, which consequently result in serious soil-borne diseases. The key reasons for intercropping *P. ginseng* were the imbalance of the rhizosphere microbial community and the increase in the abundances of soil-borne pathogens. The results of this study reveal the effects of *P. ginseng* succession cropping on the diversity and composition of soil microbial communities. The change in microbial community composition is accompanied by the change in microbial functional characteristics. The soil-borne pathogens were accumulated through cropping years of *P. ginseng*. Therefore, the transplantation seems valid to avoid succession cropping *P. ginseng*. This partly explains why *P. ginseng* planted on farmland is usually transplanted in the second or third year.

## Materials and methods

### Collection of rhizosphere soil samples

*P. ginseng* rhizosphere soil samples were collected in June 2017 from different Standardized Planting Base (See Table 1 for details of sample collection locations) using conventional methods. The soil samples were collected from the rhizosphere areas (N1/L1, N2/L2, N3/L3, N4/L4, and N5/L5) of *P. ginseng* with continuous planting for one, two, three, four, and five years, respectively. The control soil samples (NCK and LCK) were collected from the farmland and the deforestation field respectively where *P. ginseng* had not been planted. Each planting age in this study represented a different sampling location. From each sampling location, an area of 100 m^2^ was selected. In this area, five individual samples of *P. ginseng* plants were collected by the five-point sampling method. Therefore, these five biological repetitions came from the same location. *P. ginseng* roots were removed from the soil, topsoil was shakenoff, and then the soil close to the root surface was gently scraped off using cotton swabs (at least 5 g). The collected soil samples were placed in labeled sterile bags and then transported to the laboratory at low temperature. From the five samples at each sampling point, three samples were randomly taken for sequencing and further analyses.

**Table 1:** A detailed description of soil sample collection and characteristics(*n* = 5)

Sample name	Cultivation mode	Cultivation age	GPS coordinates	Bacteria		Fungi	
				Total reads	OTUs	Total reads	OTUs
NCK	Farmland	0	125.61 E,41.54 N	16,235,173	2643	11,657,696	1255
N1		1	125.61 E,41.54 N	17,742,598	2475	10,161,450	297
N2		2	125.60 E,41.54 N	17,241,033	2772	9,555,316	776
N3		3	125.61 E,41.54 N	15,425,882	2440	8,493,303	728
N4		4	125.94 E,40.98 N	15,734,231	2381	9,271,211	496
N5		5	125.94 E,40.98 N	17,114,356	2465	9,734,170	491
LCK	Forest field	0	125.80 E,41.49 N	16,475,198	2117	9,363,764	881
L1		1	125.80 E,41.49 N	15,773,793	2545	10,379,872	1138
L2		2	125.80 E,41.49 N	16,727,217	1962	10,761,135	934
L3		3	125.80 E,41.49 N	17,050,744	2478	10,665,062	718
L4		4	125.79 E,41.48 N	15,674,442	2410	9,016,774	1024
L5		5	125.79 E,41.48 N	16,904,119	2385	9,948,435	939

### DNA extraction, amplicon generation, and Illumina MiSeq sequencing

Microbial genomic DNA from *P. ginseng* rhizospheric soil samples was extracted using E.Z.N.A.® Soil DNA Kit(OMEGA, U.S.) following the operation manual. The concentration and purification of the extracted DNA was first determined by the NanoDrop 2000ultraviolet-visible spectrophotometer (Thermo Scientific, U.S.), and the quality was evaluated by electrophoresis on a 1% agarose gel.

Amplicon generation. The specific primer set 338F (5′- ACTCCTACGGGAGGCAGCAG-3′) / 806R (5′-GGACTACHVGGGTWTCTAAT-3′) [53] and ITS1F (5′-CTTGGTCATTTAGAGGAAGTAA-3′) / ITS2R (5′-GCTGCGTT CTTCATCGATGC-3′) [54] with barcode sequence were used to PCR-amplify the V3-V4 regions of the bacterial16S rRNA genes (468 bp product), and the ITS regions of fungi (300 bp product), respectively. PCR was conducted on the ABI GeneAmp® 9700 thermocycler PCR system.PCR reaction procedure was: denaturation at 95 °C for 3 min; then 27 cycles of denaturation at 95 °C for 30 s, annealing at 55 °C for 30 s, and extension at 72 °C for 45 s; and a final extension at 72 °C for 10 min. Twenty microliters of the reaction system were consisted of 5× FastPfu Buffer (4 μL), 2.5 mM dNTPs (2 μL), 5 μM single primer (0.8 μL), FastPfu Polymerase (0.4 μL),and 10 ng of template DNA. Three repeated PCR products per sample were pooled and detected by 2% agarose gel electrophoresis. The DNA fragments in the gel were purified and quantified according to the instruction manual of the AxyPrep DNA Gel Extraction Kit (Axygen Biosciences, U.S.) and QuantiFluor™-ST (Promega, U.S.), respectively.

The purified amplicons were pooled in an equimolar ratio according to the standard procedures of Majorbio Bio-Pharm Technology Co., Ltd. (Shanghai, China), and paired-end sequenced on the Illumina MiSeq platform (TruSeq™ DNA SamplePrep Kit, Illumina, U.S.). The raw reads were deposited into the NCBI Sequence Read Archive (SRA) database (Accession Number: PRJNA681095 and PRJNA681348).

### Sequencedata processing

The raw FastQ files were demultiplexed, quality filtered by Trimmomatic [55], and then merged by FLASH [56] according to the following criteria: (i)the reads were truncated at any site receiving an average quality score < 20 over a 50 bp sliding window,(ii) primers were exactly matched allowing 2 nucleotide mismatch and reads containing ambiguous bases were removed, and (iii) sequences with longer than10 bp overlap were merged according to their overlap sequence.

UPARSE(version 7.1, http://drive5.com/uparse/) [57] was used to cluster operational taxonomic units (OTUs) with 97% similarity cut off, and UCHIME (version 4.2) was used to identify and remove the chimeric sequences. The RDP Classifier (http://rdp.cme.msu.edu/) was used to analyze the taxonomy of each 16S rRNA gene and ITS sequences against the Silva (SSU132) 16S rRNA database and Unite (version 8.0) ITS database, respectively, using a confidence threshold of 70%. Then, the data were analyzed using the free online Majorbio I-Sanger Cloud Platform (www.i-sanger.com).

The indices of alpha diversity were analyzed by Mothur (version1.30.2). Forthe beta diversity analysis, the Bray-Curtis distance algorithm was used to calculate the distance between samples. The principal coordinates (PCoA) analysis was tested by ANOSIM. The Permutational Multivariate Analysis of Variance (PERMANOVA) was performed using the Bray-Curtis distance algorithm with 999 permutations.

### Statistical analysis

Alpha diversity metrics of different groups were compared by Student’s t-test. For comparative analysis of multiple groups, one-way ANOVA was conducted. The Student’s t-test was used to compare the two groups. Statistical significance was set at *p < 0.05, **p < 0.01, and ***p < 0.001.

## Supplementary Information

**Additional file 1: Figure S1.** Estimation of microbial communities by alpha diversity. A, Alpha diversity of fungal communities between NCK and LCK. B, Alpha diversity of fungal communities among LCK, L1, L2, L3, L4, and L5. Estimation of alpha diversity representing two biological replicates for the NCK samples, and three biological replicates for the each rhizospheric soil sample (*p < 0.05, **p < 0.01, ***p < 0.001).

**Additional file 2: Figure S2.** Comparison of abundance of bacteria at the phylum level. Three biological replicates for each rhizosphere soil sample (*p < 0.05, **p < 0.01, ***p < 0.001). Major contributing phyla are displayed in different colors.

**Additional file 3: Figure S3.** Comparison of bacterial abundance in the samples from two soil types at the phylum level. A, Significant differences with cultivation ages among farmland soil samples (N group). B, Significant differences with cultivation ages among forest filed soil samples (L group). Three biological replicates for each rhizospheric soil sample (*p < 0.05, **p < 0.01, ***p < 0.001).

**Additional file 4: Figure S4.** Comparison of fungal abundance at the phylum level. Three biological replicates for each rhizosphere soil sample (*p < 0.05, **p < 0.01, ***p < 0.001). Major contributing phyla are displayed in different colors.

**Additional file 5: Figure S5.** Comparison of fungal abundance in the two samples at the phylum level. Three biological replicates for each rhizosphere soil sample (*p < 0.05, **p < 0.01, ***p < 0.001).
