# Supplementary material for: Diversity and composition of the Panax ginseng rhizosphere microbiome in various cultivation modesand ages
Source: BMC Microbiol. 2021 Jan 8;21:18. doi: 10.1186/s12866-020-02081-2 (PMC7792351; doi:10.1186/s12866-020-02081-2)
Supplement: Supplementary file 1 — Additional file 1: Figure S1. Estimation of microbial communities by alpha diversity. A, Alpha diversity of fungal communities between NCK and LCK. B, Alpha diversity of fungal communities among LCK, L1, L2, L3, L4, and L5. Estimation of alpha diversity representing two biological replicates for the NCK samples, and three biological replicates for the each rhizospheric soil sample (*p < 0.05, **p < 0.01, ***p < 0.001). [file 12866_2020_2081_MOESM1_ESM.docx]

Figure S1. Estimation of microbial communities by alpha diversity. A, Alpha diversity of fungal communities between NCK and LCK. B, Alpha diversity of fungal communities among LCK, L1, L2, L3, L4, and L5. Estimation of alpha diversity representing two biological replicates for the NCK samples, and three biological replicates for the each rhizospheric soil sample (*p < 0.05, **p< 0.01, ***p < 0.001).
