# Supplementary material for: Diversity and composition of the Panax ginseng rhizosphere microbiome in various cultivation modesand ages
Source: BMC Microbiol. 2021 Jan 8;21:18. doi: 10.1186/s12866-020-02081-2 (PMC7792351; doi:10.1186/s12866-020-02081-2)
Supplement: Supplementary file 3 — Additional file 3: Figure S3. Comparison of bacterial abundance in the samples from two soil types at the phylum level. A, Significant differences with cultivation ages among farmland soil samples (N group). B, Significant differences with cultivation ages among forest filed soil samples (L group). Three biological replicates for each rhizospheric soil sample (*p < 0.05, **p < 0.01, ***p < 0.001). [file 12866_2020_2081_MOESM3_ESM.docx]

Figure S3. Comparison of bacterial abundance in the samples from two soil types at the phylum level. A, Significant differences with cultivation ages among farmland soil samples (N group). B, Significant differences with cultivation ages among forest filed soil samples (L group). Three biological replicates for each rhizospheric soil sample (*p < 0.05, **p< 0.01, ***p < 0.001).
