# Supplementary material for: Diversity and composition of the Panax ginseng rhizosphere microbiome in various cultivation modesand ages
Source: BMC Microbiol. 2021 Jan 8;21:18. doi: 10.1186/s12866-020-02081-2 (PMC7792351; doi:10.1186/s12866-020-02081-2)
Supplement: Supplementary file 4 — Additional file 4: Figure S4. Comparison of fungal abundance at the phylum level. Three biological replicates for each rhizosphere soil sample (*p < 0.05, **p < 0.01, ***p < 0.001). Major contributing phyla are displayed in different colors. [file 12866_2020_2081_MOESM4_ESM.docx]

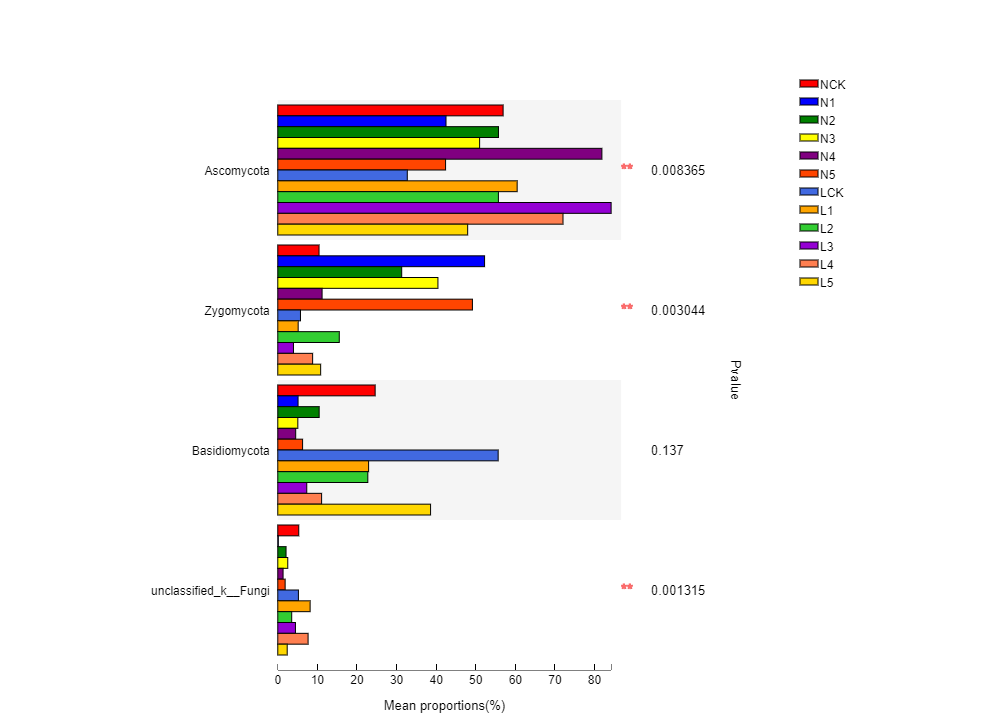


Figure S4.Comparison of fungal abundance at the phylum level. Three biological replicates for each rhizosphere soil sample (*p < 0.05, **p< 0.01, ***p < 0.001). Major contributing phyla are displayed in different colors.
