# Supplementary material for: Diversity and composition of the Panax ginseng rhizosphere microbiome in various cultivation modesand ages
Source: BMC Microbiol. 2021 Jan 8;21:18. doi: 10.1186/s12866-020-02081-2 (PMC7792351; doi:10.1186/s12866-020-02081-2)
Supplement: Supplementary file 5 — Additional file 5: Figure S5. Comparison of fungal abundance in the two samples at the phylum level. Three biological replicates for each rhizosphere soil sample (*p < 0.05, **p < 0.01, ***p < 0.001). [file 12866_2020_2081_MOESM5_ESM.docx]

Figure S5.Comparison of fungal abundance in the two samples at the phylum level. Three biological replicates for each rhizosphere soil sample (*p < 0.05, **p< 0.01, ***p < 0.001).
